# Polyphenols Suppress Intracellular Zinc Deficiency-Induced ROS Production and NLRP3 Inflammasome Activation in Microglial and Neuronal Cells

**DOI:** 10.3390/biom16060920

**Published:** 2026-06-21

**Authors:** Ayumi Matsushita, Maki Kimura, Naoko Tajima, Tsuyoshi Yamanaka, Masato Inazu

**Affiliations:** 1 Institute of Medical Science, Tokyo Medical University, 6-1-1 Shinjuku, Shinjuku-ku, Tokyo 160-8402, Japan; ayumi_matsushita@outlook.jp; 2 Department of Molecular Preventive Medicine, Tokyo Medical University, 6-1-1 Shinjuku, Shinjuku-ku, Tokyo 160-8402, Japan; be.style.m.house@gmail.com (M.K.); n.tajima@rtss.co.jp (N.T.); yamanaka0244@gmail.com (T.Y.)

**Keywords:** zinc deficiency, reactive oxygen species, NLRP3 inflammasome, polyphenols, neuroinflammation, microglia, oxidative stress, Alzheimer’s disease

## Abstract

Zinc deficiency is increasingly recognized as a risk factor for neurodegenerative diseases, yet the underlying molecular mechanisms remain incompletely understood. In this study, we investigated the impact of intracellular zinc depletion on oxidative stress and inflammasome activation in microglial (SIM-A9) and neuronal (SH-SY5Y) cell models, and evaluated the protective effects of polyphenolic compounds. Intracellular zinc chelation with the membrane-permeable chelator TPEN markedly increased reactive oxygen species (ROS) production, reduced cell viability, and upregulated the mRNA expression of NLRP3 inflammasome-related genes and pro-inflammatory cytokines. In contrast, extracellular zinc chelation had no effect, highlighting the critical role of intracellular zinc homeostasis in maintaining redox balance. Zinc supplementation significantly attenuated these responses. Among 32 polyphenols screened by DPPH radical scavenging assay, caffeic acid derivatives—chicoric acid (ChA), rosmarinic acid (RA), and caffeic acid phenethyl ester (CAPE)—exhibited the most potent antioxidant activity, surpassing that of edaravone. These compounds suppressed ROS production and differentially protected against zinc deficiency-induced cellular damage. ChA showed the strongest ROS inhibitory activity (IC50: 1.9 µM in SIM-A9), RA provided robust cytoprotection even at low concentrations, and CAPE most effectively suppressed inflammasome-related gene expression and inhibited aggregation of both Aβ1–42 and the highly neurotoxic pyroglutamate-modified variant pEAβ3–42. These findings demonstrate that intracellular zinc deficiency drives ROS-dependent upregulation of NLRP3 inflammasome-related genes, and suggest that caffeic acid derivative polyphenols may serve as complementary agents for mitigating neuroinflammatory and amyloidogenic processes relevant to Alzheimer’s disease.

## 1. Introduction

Zinc is an essential trace element that cannot be synthesized by the body, and its physiological roles are remarkably diverse. It is indispensable for maintaining the structural integrity and catalytic activity of more than 300 enzymes and is required for the structural formation of over 1000 zinc finger-type transcription factors. Consequently, zinc is recognized as a fundamental factor supporting diverse cellular functions, including gene expression regulation, cell differentiation, and metabolic homeostasis [1,2]. Furthermore, zinc contributes to antioxidant defense as a structural component of superoxide dismutase 1 (SOD1) and plays crucial roles in neurotransmitter regulation, insulin secretion, and the maturation and maintenance of immune cell function. Given these wide-ranging roles, zinc deficiency has the potential to cause dysfunction across multiple organ systems. Reported clinical manifestations of zinc deficiency include impaired memory and learning, increased susceptibility to infection, hormonal dysregulation, growth retardation, heightened oxidative stress, and delayed wound healing [3]. Of particular note, zinc also functions as a metal neurotransmitter involved in synaptic plasticity, and fluctuations in zinc concentrations within the cerebral cortex and hippocampus are thought to directly impair cognitive function [4,5].

The convergence of rising zinc deficiency prevalence and the increasing burden of neurodegenerative diseases is of particular concern in aging societies. Epidemiological data from Japan indicate that the prevalence of zinc deficiency increases markedly with age; among individuals in their 60s, 35.9% of men and 25.5% of women are classified as having pathological zinc deficiency [6]. When subclinical deficiency is included, this proportion approaches approximately 80% [6]. Meanwhile, the prevalence of Alzheimer’s disease (AD) continues to rise in parallel with population aging [7]. Taken together, these observations suggest that chronic zinc deficiency in the elderly may represent an important modifiable risk factor for neurodegenerative diseases, including AD. Consistent with this hypothesis, a meta-analysis demonstrated that serum zinc concentrations are significantly lower in AD patients compared with cognitively healthy individuals, and higher dietary zinc intake has been associated with a reduced risk of developing AD [8]. Mechanistically, zinc may modulate amyloid-β (Aβ) aggregation and deposition, and may suppress neuroinflammation, with accumulating evidence implicating disrupted zinc metabolism in the pathogenesis of AD [9].

One key mechanism through which zinc deficiency may promote neurodegeneration is the amplification of oxidative stress. As zinc is required for normal SOD1 activity, its deficiency leads to excessive ROS production and impaired antioxidant capacity [10]. It is important to note that zinc itself is a redox-inert metal and does not directly participate in Fenton-type reactions that generate hydroxyl radicals. Rather, zinc deficiency promotes ROS accumulation indirectly: by impairing SOD1 activity, zinc depletion allows superoxide to accumulate, which in turn drives Fenton and Haber–Weiss reactions mediated by redox-active metals such as copper (Cu^2+^) and iron (Fe^2+^/Fe^3+^). Furthermore, zinc normally competes with copper and iron for binding to redox-sensitive proteins, and its depletion may liberate these catalytically active metals, further amplifying ROS production. Thus, zinc should be understood as a central regulator of copper- and iron-driven oxidative chemistry, rather than a direct ROS generator. ROS accumulation in turn causes lipid peroxidation, protein oxidation, and DNA damage, thereby increasing neuronal vulnerability [11]. Furthermore, oxidative stress activates microglia—the primary innate immune cells of the central nervous system—and represents a major driver of neuroinflammation [12]. Activated microglia are known to engage the NLRP3 (NOD-like receptor family pyrin domain-containing 3) inflammasome in response to damage-associated molecular patterns (DAMPs) and pathogen-associated molecular patterns (PAMPs) [13,14]. NLRP3 inflammasome activation leads to caspase-1-dependent maturation and pyroptotic release of IL-1β and IL-18 [15,16]. Chronic release of these cytokines promotes neurotoxicity, disrupts neural circuits, and accelerates neurodegeneration. In AD, Aβ-mediated NLRP3 activation has been shown to contribute to disease progression, positioning the NLRP3 inflammasome as a compelling therapeutic target [17].

Polyphenolic compounds are plant-derived natural antioxidants with diverse biological activities, including free radical scavenging, metal chelation, anti-inflammatory effects, and modulation of intracellular signal transduction. Several polyphenols have been reported to inhibit Aβ aggregation and reduce its neurotoxic effects. Representative polyphenols such as resveratrol, catechins, and curcumin have been shown to exert multifaceted neuroprotective effects, including suppression of neuroinflammation, mitochondrial protection, and downregulation of NLRP3 inflammasome activation [18,19]. Based on these observations, polyphenols may be well-positioned to attenuate both ROS production and NLRP3 inflammasome activation that are amplified under zinc-deficient conditions.

In the present study, we aimed to elucidate the impact of intracellular zinc deficiency on ROS production and NLRP3 inflammasome-related gene expression in microglial and neuronal cell models, and to investigate the protective effects of caffeic acid derivative polyphenols against zinc deficiency-induced oxidative stress and neuroinflammatory responses.

## 2. Materials and Methods

### 2.1. Cell Culture

The mouse microglial cell line SIM-A9 (Applied Biological Materials Inc., Richmond, BC, Canada) and the human neuroblastoma cell line SH-SY5Y (DS Pharma Biomedical Co., Ltd., Osaka, Japan) were maintained in RPMI-1640 medium (FUJIFILM Wako, Osaka, Japan) supplemented with 10% fetal bovine serum (FBS; Biosera, Cholet, France) and 1% penicillin–streptomycin (FUJIFILM Wako, Osaka, Japan) at 37 °C in a humidified atmosphere containing 5% CO_2_. Culture medium was replaced every 2–3 days.

### 2.2. Screening of Polyphenols by DPPH Radical Scavenging Assay

A total of 32 polyphenolic compounds were dissolved in dimethyl sulfoxide (DMSO; FUJIFILM Wako, Osaka, Japan) to a final concentration of 25 µM. Radical scavenging activity was evaluated using 1,1-diphenyl-2-picrylhydrazyl (DPPH; Tokyo Chemical Industry, Tokyo, Japan). DPPH was prepared at 100 µM in methanol and added to 48-well plates containing each test compound. After incubation for 1 h at room temperature in the dark, absorbance at 517 nm was measured using a microplate reader (FilterMax F5; Molecular Devices, San Jose, CA, USA). Radical scavenging activity was calculated as the percentage inhibition of absorbance relative to the DMSO vehicle control.

### 2.3. Measurement of Intracellular ROS

Intracellular ROS levels were measured using the fluorescent probe 2′,7′-dichlorodihydrofluorescein diacetate (DCFH-DA; Cell Biolabs, San Diego, CA, USA). SIM-A9 and SH-SY5Y cells were seeded in 96-well plates at densities of 1 × 10^4^ and 2 × 10^4^ cells/well, respectively. Cells were incubated with 100 µM DCFH-DA for 30 min, washed with Hank’s balanced salt solution (HBSS), and subsequently incubated in Opti-MEM (Thermo Fisher Scientific, Waltham, MA, USA). Fluorescence was measured every 10 min for up to 24 h at 37 °C (Ex 485 nm/Em 535 nm). To induce intracellular zinc deficiency, cells were treated with the membrane-permeable zinc chelator N,N,N′,N′-tetrakis(2-pyridinylmethyl)-1,2-ethanediamine (TPEN; 0.01–10 µM; Tokyo Chemical Industry, Tokyo, Japan). Extracellular zinc chelation was performed using CaEDTA (Tokyo Chemical Industry, Tokyo, Japan) or ZnEDTA (50 µM each; Tokyo Chemical Industry, Tokyo, Japan). Zinc supplementation experiments employed ZnCl_2_ (0.156–10 µM; Tokyo Chemical Industry, Tokyo, Japan). For polyphenol experiments, caffeic acid (CA; Tokyo Chemical Industry, Tokyo, Japan), CAPE (Tokyo Chemical Industry, Tokyo, Japan), ChA (Tokyo Chemical Industry, Tokyo, Japan), and RA (FUJIFILM Wako, Osaka, Japan) (1.5625–10 µM) were applied 1 h prior to stimulation with TPEN (3–5 µM) or hydrogen peroxide (H_2_O_2_; 1 mM).

### 2.4. Cell Viability Assay

Cell viability was assessed using the PrestoBlue cell viability reagent (Invitrogen, Waltham, MA, USA). Cells were seeded in 96-well plates and pretreated with polyphenols (1.5625–100 µM) for 1 h, followed by exposure to TPEN (5 µM) or H_2_O_2_ (1 mM) for 4 h. PrestoBlue reagent was then added and incubated for 30 min. Fluorescence was measured at Ex 535 nm/Em 595 nm. Cell viability was expressed as a percentage relative to the vehicle control.

### 2.5. Quantitative RT-PCR

SIM-A9 cells were seeded in 6-well plates and pretreated with polyphenols for 1 h, followed by TPEN (0.3–30 µM) for 4 h. For zinc supplementation experiments, ZnCl_2_ was added prior to TPEN treatment. Total RNA was extracted using ISOGEN reagent (Nippon Gene, Tokyo, Japan) followed by purification with the Direct-zol RNA Kit (Zymo Research, Irvine, CA, USA). RNA concentrations were determined using a NanoDrop 2000c spectrophotometer (Thermo Fisher Scientific, Waltham, MA, USA). Quantitative RT-PCR was performed using the TaqMan RNA-to-CT 1-Step Kit (Applied Biosystems, Foster City, CA, USA) with TaqMan Gene Expression Assays targeting NLRP3, IL-1β, IL-18, TNF-α, and B2M (reference gene). Amplification was carried out using a LightCycler 96 system (Roche Diagnostics, Basel, Switzerland). Relative gene expression was calculated by the comparative threshold-cycle (ΔΔCt) method.

### 2.6. Detection of Aβ Aggregates Using Thioflavin T and Transmission Electron Microscopy

Aggregation of Aβ1–42 and pEAβ3–42 (PEPTIDE INSTITUTE, INC., Osaka, Japan) was assessed using the SensoLyte Thioflavin T β-Amyloid (1–42) Aggregation Kit (AnaSpec Inc., Fremont, CA, USA) according to the manufacturer’s protocol. In brief, 5 µL of 2 mM thioflavin T (ThT) and 0.5 µL of each polyphenol or ALZ-801-related compounds were added to wells of a Corning 96 Well Half-Area Microplate and mixed with 44.5 µL of Aβ1–42 or pEAβ3–42 solution. Final concentrations were 25 µM for both peptides and 6.25–100 µM for each polyphenol. Fluorescence (Ex/Em = 440/484 nm) was measured every 5 min for 3 h at 37 °C with 15 s shaking between readings using a FilterMax F5 microplate reader (Molecular Devices, San Jose, CA, USA).

For transmission electron microscopy (TEM), amyloid samples were applied to 400-mesh carbon-coated copper grids that had been glow-discharged prior to use and adsorbed for 1 min. Excess solution was blotted with filter paper. Grids were briefly rinsed with deionized water and negatively stained with 2% (*w*/*v*) uranyl acetate for 30–60 s. After removal of excess stain, grids were air-dried at room temperature. At least three independent grids were prepared per sample. Electron micrographs were acquired using a JEM-1400Flash transmission electron microscope (JEOL Ltd., Tokyo, Japan) operated at 100 kV over a magnification range of 10,000×–100,000×.

### 2.7. Statistical Analysis

All data are presented as mean ± standard deviation (SD). For comparisons among multiple groups, one-way analysis of variance (ANOVA) followed by Dunnett’s multiple comparison test was used. Comparisons between two groups were performed using Student’s *t*-test. A *p*-value < 0.05 was considered statistically significant. These statistical analyses were performed using GraphPad Prism 11 software (GraphPad, San Diego, CA, USA).

## 3. Result

### 3.1. Identification of Potent Antioxidant Polyphenols by DPPH Screening

**Figure 1:**
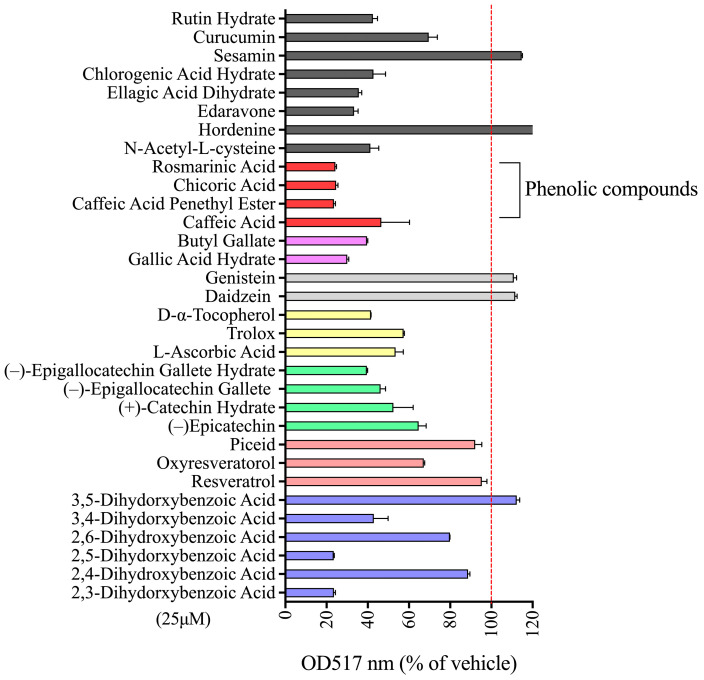
DPPH Radical Scavenging Activity of 32 Polyphenolic Compounds. The DPPH radical scavenging activity of 32 polyphenolic compounds (final concentration: 25 µM) was evaluated. The inhibitory effect of each polyphenolic compound was calculated by setting the absorbance at 517 nm in the vehicle control (DMSO) to 100%. Four phenolic compounds (red columns)—caffeic acid (CA), caffeic acid phenethyl ester (CAPE), chicoric acid (ChA), and rosmarinic acid (RA)—exhibited high activity, and these four compounds were selected as the subjects of the experiment. Each plot is presented as the mean ± SD (*n* = 4).

**Figure 2:**
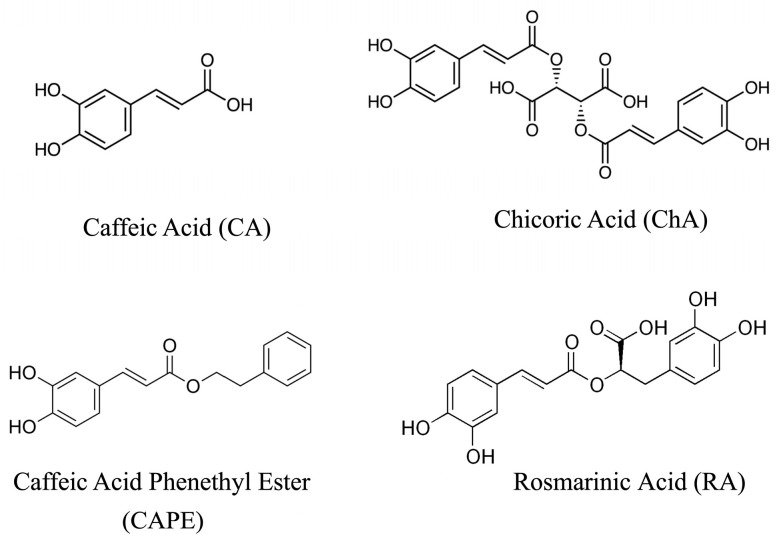
Structural formula of phenolic compounds. 3-(3,4-Dihydroxyphenyl)-2-propenoic acid (caffeic acid, CA), (2R,3R)-2,3-Bis{[(2E)-3-(3,4-dihydroxyphenyl)prop-2-enoyl]oxy}butanedioic acid (chicoric acid, ChA), 2-Phenylethyl (2E)-3-(3,4-dihydroxyphenyl)prop-2-enoate (caffeic acid phenethyl ester, CAPE) and (2R)-3-(3,4-Dihydroxyphenyl)-2-{[(2E)-3-(3,4-dihydroxyphenyl)prop-2-enoyl]oxy}propanoic acid (rosmarinic acid, RA).

To identify candidate compounds with strong antioxidant capacity, we first evaluated the radical scavenging activity of 32 polyphenolic compounds using the DPPH assay (Figure 1). Among all tested compounds, four caffeic acid-related compounds—CA, CAPE, ChA, and RA—exhibited the highest radical scavenging activity. Notably, CAPE, ChA, and RA each showed stronger activity than edaravone, a clinically used free radical scavenger, indicating potent antioxidant properties. These four compounds were therefore selected for all subsequent analyses. The structural formulas of the four compounds are shown in Figure 2.

### 3.2. Intracellular Zinc Deficiency Selectively Induces ROS Production

**Figure 3:**
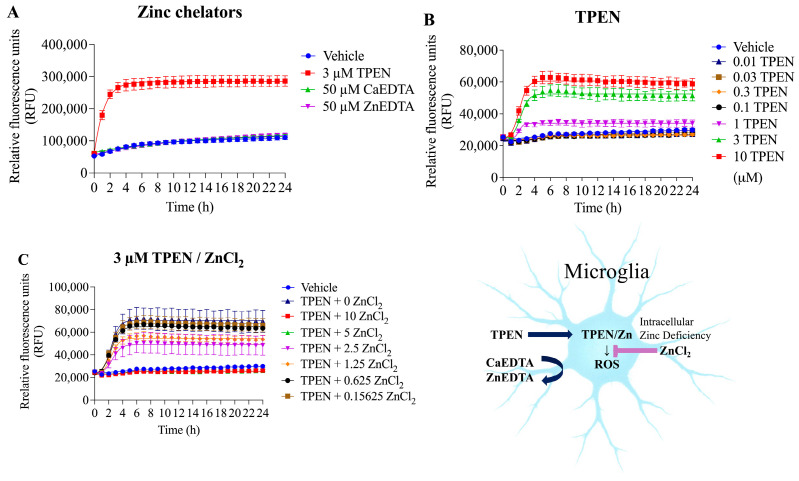
Effects of Intracellular and Extracellular Zinc Chelators on Intracellular ROS Production in SIM-A9 Cells. (**A**) Changes in intracellular ROS production over time following treatment of SIM-A9 cells with the intracellular zinc chelator TPEN (3 µM), the extracellular zinc chelator CaEDTA (50 µM), and the negative control ZnEDTA (50 µM). The vehicle was 1% DMSO. (**B**) Changes in intracellular ROS production over time following treatment of SIM-A9 cells with the intracellular zinc chelator TPEN at final concentrations ranging from 0.01 to 10 µM. (**C**) Effect of zinc chloride (0.15625–10 µM) on intracellular ROS production induced by 3 µM TPEN. Zinc chloride was administered 1 h prior to TPEN treatment. Each plot is shown as the mean ± SD (*n* = 4).

To determine whether zinc depletion drives oxidative stress, intracellular ROS levels were measured following zinc chelation in SIM-A9 microglial cells. Treatment with the membrane-permeable chelator TPEN significantly increased ROS production in a concentration-dependent manner (Figure 3A,B). In contrast, extracellular zinc chelation with CaEDTA, or the negative control ZnEDTA, did not induce ROS generation, clearly demonstrating that intracellular—rather than extracellular—zinc depletion is the critical trigger of oxidative stress (Figure 3A). Furthermore, pre-supplementation with ZnCl_2_ markedly suppressed TPEN-induced ROS production in a dose-dependent fashion (Figure 3C), confirming the specificity of the TPEN effect.

### 3.3. Polyphenols Suppress TPEN- and H_2_O_2_-Induced ROS Production

#### 3.3.1. Effects in SIM-A9 Cells

**Figure 4:**
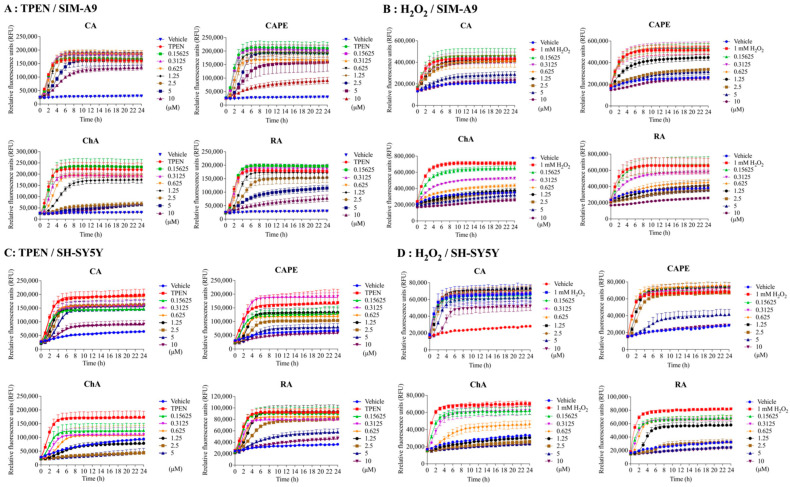
Effects of Polyphenolic Compounds on TPEN- and H_2_O_2_-Induced Intracellular ROS Production in SIM-A9 (**A**,**B**) and SH-SY5Y (**C**,**D**) Cells. Changes in intracellular ROS production over time following pretreatment with the polyphenolic compounds caffeic acid (CA), caffeic acid phenethyl ester (CAPE), chicoric acid (ChA), and rosmarinic acid (RA) at concentrations of 0.15625–10 µM, followed by treatment with 3 µM TPEN (**A**,**C**) and 1 mM H_2_O_2_ (**B**,**D**). Each plot is shown as the mean ± SD (*n* = 4).

Pretreatment with all four polyphenols significantly reduced TPEN (3 µM)-induced ROS production in SIM-A9 microglial cells, in the order ChA > CAPE > RA > CA (Figure 4A). All four compounds also exhibited strong inhibitory effects against H_2_O_2_ (1 mM)-induced ROS production, in the order ChA > RA > CAPE > CA (Figure 4B). The respective IC_50_ values are summarized in Table 1.

#### 3.3.2. Effects in SH-SY5Y Cells

Consistent effects were observed in the neuronal SH-SY5Y cell line. All four polyphenols inhibited TPEN (5 µM)– and H_2_O_2_ (1 mM)-induced ROS production (Figure 4C,D), with the inhibitory order against TPEN being ChA > CAPE > RA > CA, and against H_2_O_2_ being ChA > RA > CAPE > CA. These results indicate that the antioxidant activity of these caffeic acid derivative polyphenols is broadly applicable across cell types (Table 1).

### 3.4. Polyphenols Exhibit Differential Cytoprotective Effects

**Figure 5:**
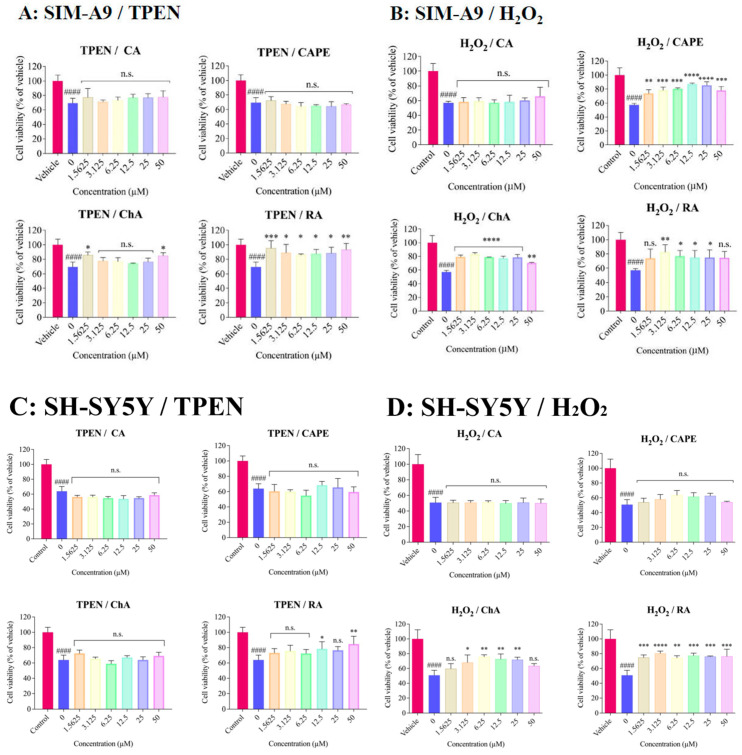
Effects of Polyphenolic Compounds on TPEN- and H_2_O_2_-Induced Cell Death in SIM-A9 (**A**,**B**) and SH-SY5Y (**C**,**D**) Cells. Cells were pretreated with polyphenolic compounds at various concentrations (1.5625–100 µM) for 1 h, followed by the addition of 5 µM TPEN; cell viability was assessed 4 h later. Viability at each concentration was calculated relative to the vehicle control (100%). Data are presented as mean ± SD (*n* = 4). A *t*-test was performed to compare the Vehicle group with the TPEN group (0). ####: *p* < 0.0001. To test the effects of the polyphenolic compounds, an ANOVA was performed, followed by Dunnett’s multiple comparison test, with a significance level of 5%. *: *p* < 0.05, **: *p* < 0.01, ***: *p* < 0.001, ****: *p* < 0.0001, n.s.: not significant.

The effects of polyphenols on cell viability under oxidative stress were assessed in both SIM-A9 and SH-SY5Y cells. TPEN treatment significantly reduced cell viability in SIM-A9 cells; this effect was effectively counteracted by ChA and RA, with RA demonstrating protective effects even at low concentrations (Figure 5A). In contrast, CA and CAPE did not exert significant protective effects against TPEN-induced cytotoxicity under these conditions. Under H_2_O_2_ stress, ChA, RA, and CAPE each improved cell viability, whereas CA showed minimal effects (Figure 5B). Similar patterns were observed in SH-SY5Y neuronal cells (Figure 5C,D). Collectively, these findings indicate that polyphenols possess distinct cytoprotective profiles, with RA and ChA demonstrating the most robust protective effects across cell types and stress conditions.

### 3.5. Intracellular Zinc Deficiency Upregulates NLRP3 Inflammasome-Related Gene Expression

**Figure 6:**
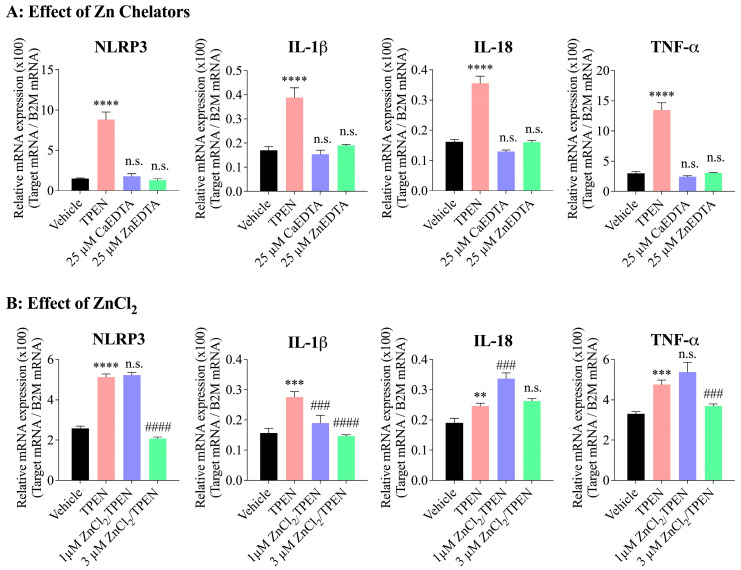
Effects of Intracellular and Extracellular Zinc Chelators on the mRNA Expression of NLRP3, IL-1β, IL-18 and TNF-α in SIM-A9 Cells. SIM-A9 cells were treated with 3 µM TPEN, 25 µM ZnEDTA, and 25 µM CaEDTA for 4 h, and the mRNA expression levels of NLRP3, IL-1β, IL-18, and TNF-α were analyzed by RT-PCR (**A**). The obtained signals were normalized against B2M expression levels and quantified as relative expression levels. Data are presented as mean ± SD (*n* = 4). Statistical analysis was performed using ANOVA followed by Dunnett’s multiple comparison test, with a significance level of 5%. ****: *p* < 0.0001; n.s.: not significant. SIM-A9 cells were pretreated with 1 or 3 µM ZnCl_2_ and treated with 3 µM TPEN for 4 h; mRNA expression of NLRP3, IL-1β, IL-18, and TNF-α was analyzed by RT-PCR (**B**). The signals obtained were normalized against B2M expression levels and quantified as relative expression levels. Data are presented as mean ± SD (*n* = 4). Comparisons between the Vehicle group and the TPEN group were performed using a *t*-test. **: *p* < 0.01, ***: *p* < 0.001, ****: *p* < 0.0001. To test the effects of zinc supplementation, we performed an ANOVA followed by Dunnett’s multiple comparison test, setting the significance level at 5%. ###: *p* < 0.001, ####: *p* < 0.0001, n.s.: not significant.

To determine whether intracellular zinc deficiency induces an inflammatory response, mRNA expression of NLRP3 inflammasome-related genes was analyzed in SIM-A9 cells. TPEN treatment significantly upregulated the expression of NLRP3, IL-1β, IL-18, and TNF-α. In contrast, neither the extracellular chelator CaEDTA nor the negative control ZnEDTA induced any change in gene expression (Figure 6A), confirming that transcriptional upregulation of inflammasome-related genes requires intracellular zinc depletion. TPEN-induced upregulation of these genes was suppressed by zinc supplementation in a concentration-dependent manner, with a clear inhibitory effect observed at 3 µM ZnCl_2_ (Figure 6B). It should be noted that the present study assessed mRNA expression only; protein-level assays (e.g., caspase-1 activation, IL-1β secretion, ASC speck formation, or gasdermin D cleavage) were not performed. Therefore, the current evidence supports transcriptional induction of inflammasome-related genes rather than definitive inflammasome activation.

**Figure 7:**
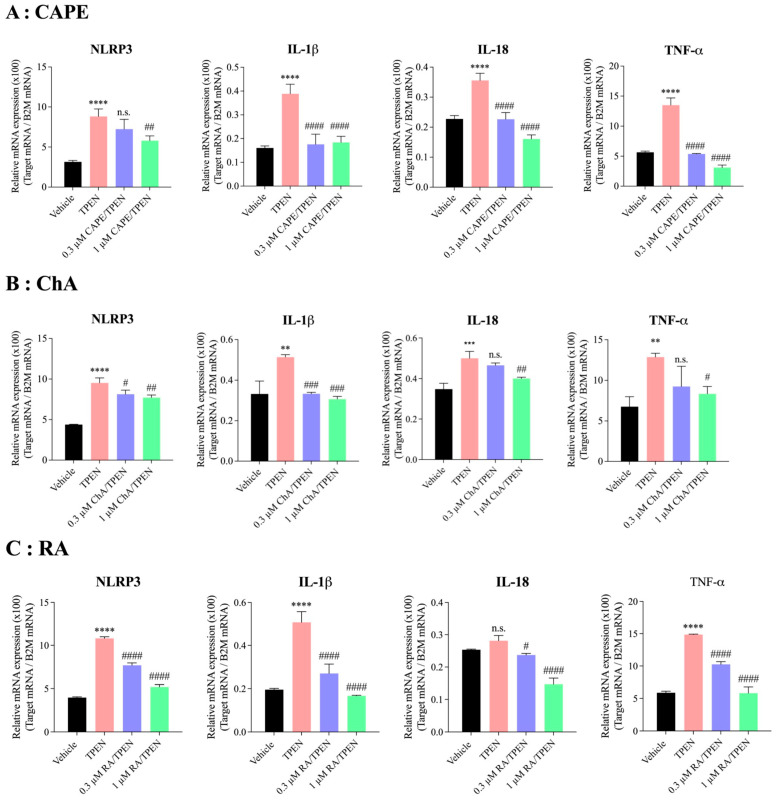
Effects of Polyphenolic Compounds on TPEN-Induced Upregulation of NLRP3 Inflammasome-Related mRNA Expression in SIM-A9 Cells. SIM-A9 cells were pretreated with 0.3 or 1 µM CAPE (**A**), ChA (**B**), or RA (**C**) for 1 h, followed by treatment with 3 µM TPEN for 4 h; mRNA expression levels of NLRP3, IL-1β, IL-18, and TNF-α were then analyzed by RT-PCR. The obtained signals were normalized against B2M expression levels and quantified as relative expression levels. Data are presented as mean ± SD (*n* = 4). Comparisons between the Vehicle and TPEN groups were performed using a *t*-test. **: *p* < 0.01, ***: *p* < 0.001, ****: *p* < 0.0001, ns: not significant. To test the effects of polyphenolic compounds, ANOVA was performed followed by Dunnett’s multiple comparison test, with a significance level of 5%. #: *p* < 0.05, ##: *p* < 0.01, ###: *p* < 0.001, ####: *p* < 0.0001, n.s.: not significant.

Pretreatment with CAPE, ChA, and RA each significantly suppressed the TPEN-induced upregulation of all four genes. Among these, CAPE exhibited the most potent inhibitory effect, restoring expression levels close to those of vehicle-treated controls (Figure 7). These results demonstrate that caffeic acid derivative polyphenols can effectively suppress zinc deficiency-induced upregulation of NLRP3 inflammasome-related genes.

### 3.6. CAPE Potently Inhibits Aβ Aggregation

Given that Aβ1–42 aggregation and fibril formation are central to AD pathology, we investigated the ability of the four polyphenols to inhibit this process. CAPE inhibited Aβ1–42 aggregation in a concentration-dependent manner; notably, in the ThT fluorescence kinetics assay at higher concentrations (100 µM), the fluorescence signal initially rose and then declined below the level of the vehicle control over the time course of the experiment, suggesting a possible disassembly effect on nascent or early-stage aggregates. However, as these observations were obtained from the kinetic aggregation assay rather than from experiments using pre-formed fibrils, it remains to be determined whether CAPE can actively disassemble mature Aβ fibrils. Direct evaluation using pre-formed fibril preparations will be an important objective of future studies. No inhibitory effect was observed for CA, ChA, or RA under these conditions.

**Figure 8:**
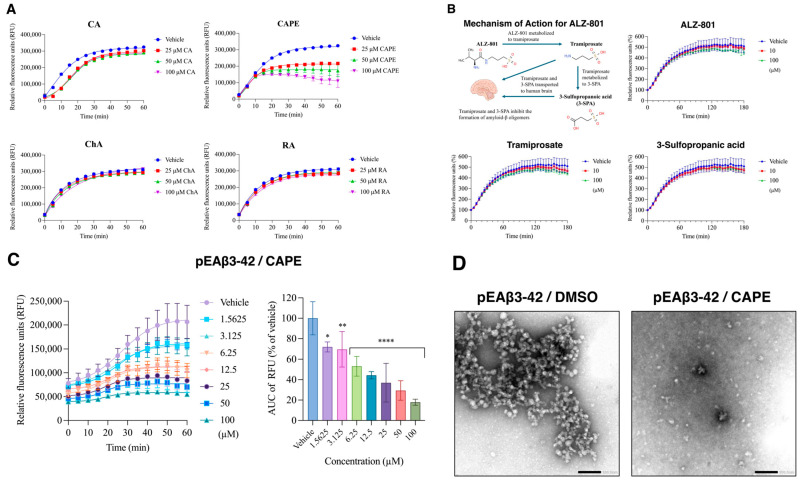
Effects of Polyphenolic Compounds and ALZ-801-related compounds on Aβ aggregation. (**A**) Effects of polyphenolic compounds on Aβ1–42 aggregation kinetics measured by ThT fluorescence assay. Time-dependent aggregation curves are shown for caffeic acid (CA), caffeic acid phenethyl ester (CAPE), chicoric acid (ChA), and rosmarinic acid (RA) at the indicated concentrations (6.25–100 μM). Data are expressed as relative fluorescence units (RFU). Data are presented as mean ± SD (*n* = 4). (**B**) Proposed mechanism of action for ALZ-801 and its metabolites in inhibiting Aβ aggregation (schematic). ALZ-801 is metabolized to tramiprosate, which is further converted to 3-sulfopropanoic acid (3-SPA). Both tramiprosate and 3-SPA are suggested to interfere with Aβ oligomer formation. Right panels show the effects of ALZ-801, tramiprosate, and 3-SPA on Aβ1–42 aggregation kinetics at indicated concentrations (10 and 100 μM), assessed by ThT fluorescence. Data are presented as mean ± SD (*n* = 4). (**C**) Inhibitory effect of CAPE on pEAβ3-42 aggregation. Left: concentration-dependent suppression of aggregation kinetics (1.5625–100 μM). Right: quantification of aggregation based on area under the curve (AUC), expressed as percentage relative to vehicle control. Data represent mean ± SD. Statistical significance is indicated (* *p* < 0.05, ** *p* < 0.01, **** *p* < 0.0001). (**D**) Transmission electron microscopy (TEM) images of pEAβ3-42 aggregates formed. Left: 25 μM pEAβ3-42 with DMSO control showing abundant fibrillar/aggregated structures. Right: co-treatment with 25 μM pEAβ3-42 and 25 μM CAPE showing reduced aggregate formation. Each image includes a 100 nm scale bar.

ALZ-801 (valiltramiprosate) is an investigational oral prodrug of tramiprosate currently under clinical development for AD, designed to prevent the formation of toxic Aβ oligomers [20]. Its active metabolite, 3-sulfopropanoic acid (3-SPA), is believed to mediate this effect. In comparative experiments, ALZ-801, tramiprosate, and 3-SPA failed to show significant inhibitory effects at 100 µM (Figure 8B), the concentration at which CAPE demonstrated clear inhibitory activity, suggesting superior potency of CAPE in this assay system.

pEAβ3–42 (pyroglutamate-modified Aβ starting at position 3) is a highly aggregation-prone and neurotoxic Aβ variant that accumulates early in AD brains and is considered a key driver of amyloid pathology [21]. Using the ThT fluorescence assay and TEM, we demonstrated that CAPE inhibits pEAβ3–42 aggregation in a concentration-dependent manner, with significant inhibition at concentrations as low as 1.5625 µM (Figure 8C). TEM observations confirmed these findings: while vehicle-treated samples contained abundant high-density oligomeric aggregates, only sparsely distributed microparticles were detected in the presence of 25 µM CAPE (Figure 8D). Collectively, these data identify CAPE as a potent inhibitor of both Aβ1–42 and pEAβ3–42 aggregation.

### 3.7. Functional Differentiation Among Caffeic Acid Derivative Polyphenols

Taken together, the four caffeic acid derivatives display distinct but complementary functional profiles, as detailed in the discussion below.

## 4. Discussions

In the present study, we evaluated the DPPH radical scavenging activity of 32 polyphenolic compounds and identified four caffeic acid derivatives—CA, CAPE, ChA, and RA—as the most potent antioxidants. All four share a catechol-containing caffeic acid core; however, structural differences in their side chains account for divergent physicochemical properties. CA and CAPE each possess a single phenolic group, while ChA and RA possess two, conferring elevated electron-donating capacity and higher radical scavenging potency. Although CAPE also contains only a single phenolic group, its hydrophobic phenethyl side chain confers high membrane permeability (XLOGP3 = 4.15; TPSA = 66.76 Å^2^), potentially enabling greater intracellular accumulation and thereby stronger intracellular antioxidant effects. These observations indicate that both aromatic substitution pattern and side chain physicochemical properties jointly determine antioxidant activity.

ROS measurements using DCFH-DA clearly demonstrated that the intracellular zinc chelator TPEN induced a rapid, concentration-dependent increase in ROS, which was suppressed by exogenous zinc supplementation. Crucially, neither CaEDTA (an extracellular zinc chelator with no cell permeability) nor ZnEDTA (a zinc-loaded control that does not deplete intracellular zinc) induced ROS generation. These results strongly suggest that intracellular—not extracellular—zinc depletion is a key trigger for cellular oxidative stress. Several limitations of the TPEN-based zinc depletion model warrant discussion. First, TPEN induces abrupt and near-complete intracellular zinc depletion, which may not accurately recapitulate the gradual and partial zinc dyshomeostasis that occurs in aging or neurodegenerative disease. Second, TPEN is not entirely zinc-selective; given its high affinity for divalent metal ions (log K: Zn^2+^ ~15.6, Cu^2+^ ~20.4, Fe^2+^ ~14.6), TPEN may simultaneously chelate intracellular copper and iron under certain conditions. The intracellular fate of TPEN-bound zinc is also relevant: the TPEN–Zn^2+^ complex is membrane-permeant and may be exported from the cell or redistributed within intracellular compartments, potentially affecting metal homeostasis beyond simple zinc depletion. Although the ZnCl_2_ rescue experiments and the absence of ROS induction by extracellular chelators (CaEDTA, ZnEDTA) provide strong evidence that intracellular zinc depletion is a primary driver of the observed effects, the possible contribution of off-target copper or iron chelation cannot be entirely excluded. Future studies using more selective intracellular zinc fluorescent sensors (e.g., FluoZin-3) or genetic zinc depletion strategies (e.g., ZIP transporter knockdown) would further strengthen the specificity of these conclusions. It is also important to note that chronic extracellular zinc deficiency can secondarily reduce intracellular zinc levels by diminishing cellular uptake. In this regard, the age-related decline in zinc absorption capacity [22] may create a state of chronic intracellular zinc insufficiency that progressively compromises antioxidant defenses and promotes oxidative stress.

SOD1 is a zinc-dependent metalloenzyme whose activity is markedly reduced by zinc deficiency [23]. TPEN-induced zinc depletion most likely inhibits SOD1 activity, leading to insufficient superoxide dismutation and consequent ROS accumulation. It should be clarified that zinc is a redox-inert metal that does not itself generate ROS through Fenton-type chemistry. Rather, the role of zinc in oxidative stress is regulatory: zinc deficiency impairs SOD1-mediated superoxide clearance, allowing superoxide to drive Fenton and Haber–Weiss reactions catalyzed by redox-active copper and iron ions. Moreover, zinc normally competes with copper and iron for binding to redox-sensitive proteins; zinc depletion may therefore liberate these catalytically active metals, further amplifying ROS generation. This mechanistic framework positions zinc as an indirect but critical regulator of copper- and iron-driven oxidative stress, which is consistent with the observed ROS accumulation upon intracellular zinc chelation and its reversal by zinc supplementation. Among the polyphenols tested, CAPE exhibited relatively stronger inhibition of TPEN-induced compared with H_2_O_2_-induced ROS. This may be explained by the ability of CAPE to activate the Keap1/Nrf2 pathway [24] and to enhance SOD activity [25], potentially compensating for the SOD1 dysfunction caused by intracellular zinc deficiency. Because superoxide lies upstream of hydrogen peroxide in the ROS cascade and its removal involves multiple enzymatic steps, the inhibitory effect on TPEN-induced ROS may be more pronounced than on H_2_O_2_-induced ROS.

In cell viability experiments, TPEN-induced cytotoxicity in microglial cells was significantly attenuated by ChA and RA. Notably, RA showed a stable protective effect even at low concentrations in both cell types. This is consistent with previous clinical evidence that RA supplementation improves cognitive function in AD patients [26], and supports its feasibility as a therapeutic agent for neurodegenerative conditions. The limited cytoprotective effects of CA and CAPE against TPEN-induced cytotoxicity highlight compound-specific functional differences that may reflect distinct intracellular targets or mechanisms.

Intracellular zinc deficiency induced by TPEN strongly upregulated NLRP3, IL-1β, IL-18, and TNF-α mRNA expression, whereas extracellular zinc chelation had no effect. These findings are consistent with the established mechanism by which ROS activates NLRP3 inflammasome-related signaling [13] and support reports that persistent inflammatory gene upregulation may exacerbate neurodegenerative diseases [27]. It is important to note, however, that the present study assessed only mRNA expression levels; protein-level and functional inflammasome assays—such as caspase-1 activation, IL-1β secretion, ASC speck formation, or gasdermin D cleavage—were not performed. The current evidence should therefore be interpreted as transcriptional induction of inflammasome-related genes rather than definitive inflammasome activation. Future studies incorporating such functional assays will be required to clarify whether full inflammasome assembly and canonical activation occur under these conditions. Among the three active polyphenols tested, CAPE exhibited the most potent suppression of inflammasome-related gene expression, with significant inhibitory activity at concentrations as low as 0.3 µM. This potency is likely attributable to the high lipophilicity of CAPE, which enhances its cellular permeability and intracellular accumulation relative to ChA and RA. CAPE is additionally known to inhibit NF-κB nuclear translocation [28], which may further contribute to its suppression of inflammatory signaling cascades.

The inhibitory activity of CAPE against Aβ aggregation is of potential relevance to Alzheimer’s disease research, though the translational implications remain preliminary at this stage, as all experiments were conducted in cell-free or in vitro systems. ALZ-801, an oral prodrug of tramiprosate currently in Phase III clinical development for AD, acts by preventing formation of soluble Aβ oligomers, which are considered more neurotoxic than fibrillar aggregates [29,30,31,32]. Unlike antibody-based therapies that target established plaques, ALZ-801 acts upstream to prevent toxic oligomer assembly. In our assay system, neither ALZ-801 nor its active metabolites showed significant inhibitory effects at 100 µM, whereas CAPE achieved clear inhibition at equivalent concentrations. In the ThT fluorescence kinetics assay at 100 µM, CAPE also caused the ThT signal to decline below baseline after an initial transient rise, suggesting a possible effect on nascent or early-stage aggregates. Whether CAPE can actively disassemble mature pre-formed Aβ fibrils remains to be determined using pre-formed fibril preparations, and this represents an important direction for future investigation. It should be noted, however, that ThT fluorescence assays can be susceptible to inner filter effects or direct interactions between test compounds and the dye, potentially confounding interpretation. To address this concern, we measured the UV-vis absorption spectrum of 100 µM CAPE and confirmed that its absorbance in the ThT measurement wavelength range (Ex 440 nm/Em 484 nm) was negligible (absorbance ≈ 0), demonstrating the absence of significant inner filter effects. Furthermore, we directly assessed the quenching effect of 100 µM CAPE on ThT fluorescence in the absence of Aβ peptides; the fluorescence intensity of ThT with CAPE (ThT-CAPE: ~34,000 RFU) was essentially identical to that of the DMSO vehicle control (ThT-DMSO: ~35,000 RFU), confirming that CAPE does not quench ThT fluorescence. These data are presented as Supplementary Materials. Together, these controls support the validity of the Aβ aggregation inhibition data. Furthermore, CAPE effectively inhibited aggregation of pEAβ3–42, a particularly pathogenic Aβ species that is believed to act as a nucleation core for amyloid seeding [21]. The ability of CAPE to target this upstream seeding event suggests a potentially promising mechanism, warranting further investigation in cellular and in vivo models.

Taken together, these caffeic acid derivatives exhibit complementary neuroprotective profiles: ChA as an antioxidant/ROS scavenger, RA as a cytoprotective agent, and CAPE as an anti-inflammatory agent and Aβ aggregation inhibitor. A combinatorial approach targeting multiple pathological nodes may therefore offer enhanced benefit. The substantial limitations of the current in vitro study are noted above, and validation in primary cell systems and in vivo models will be essential before any therapeutic conclusions can be drawn. Several limitations of the present study should be acknowledged. First, all experiments were conducted using immortalized cell lines (SIM-A9 and SH-SY5Y), which may not fully recapitulate the biology of primary neurons or microglia in vivo; accordingly, the physiological and translational relevance of these findings to human neurodegenerative disease remains to be established. Second, no in vivo validation was performed, and the therapeutic implications for Alzheimer’s disease described here should be regarded as preliminary; definitive conclusions would require demonstration of recovery of neuronal function, preservation of synaptic integrity, attenuation of Aβ toxicity, and BBB permeability studies in relevant animal models. Third, the Aβ aggregation inhibition data for CAPE were obtained by ThT fluorescence assay; complementary structural approaches such as dot blot assay or atomic force microscopy (AFM) would provide additional resolution of aggregate morphology and further substantiate these findings. Fourth, while in silico prediction using SwissADME indicates that CAPE is BBB-permeant (BBB permeant: Yes; consensus logP ~3.2; GI absorption: High; TPSA: 66.76 Å^2^), in vivo pharmacokinetic and efficacy studies have not been conducted, and the neurotoxicity of CAPE-derived Aβ aggregation products in vivo remains to be evaluated. Fifth, TEM analysis of Aβ aggregation was primarily qualitative and based on representative images; quantitative image analysis across multiple fields would strengthen conclusions regarding aggregate morphology. Sixth, the statistical analyses relied on biological replicates specified in the figure legends; additional independent experiments would further support the robustness of the findings. Because polyphenolic compounds can modulate multiple nodes of this pathological cascade, they represent promising candidates for future investigation as preventive or adjunctive agents for neurodegenerative diseases, subject to validation in more physiologically relevant experimental systems.

## 5. Conclusions

This study demonstrates that intracellular zinc deficiency promotes ROS-dependent transcriptional upregulation of NLRP3 inflammasome-related genes and neuroinflammatory responses in both microglial and neuronal cell models. Among 32 polyphenolic compounds screened, four caffeic acid derivatives—CA, CAPE, ChA, and RA—exhibited potent antioxidant activity, with each compound displaying a distinct functional profile: ChA as the most effective ROS scavenger, RA as the most robust cytoprotective agent, and CAPE as the most potent suppressor of inflammasome-related gene expression and inhibitor of Aβ aggregation. These complementary properties suggest that targeting both zinc homeostasis and oxidative stress pathways with polyphenolic agents may represent a promising strategy worthy of further investigation for the prevention and treatment of neurodegenerative diseases, including Alzheimer’s disease. As all experiments were conducted in vitro using immortalized cell lines, future studies incorporating primary cells and in vivo models will be essential to validate these findings and establish translational relevance.

## Figures and Tables

**Table 1:** The IC_50_ values of polyphenolic compounds against TPEN- and H_2_O_2_-induced intracellular ROS production in SIM-A9 and SH-SY5Y cells.

	ChA	CAPE	RA	CA	Order
SIM-A9/TPEN	1.9	5.4	7.4	>10	ChA > CAPE > RA > CA
SIM-A9/H_2_O_2_	0.4	1.9	0.5	4.5	ChA > RA > CAPE > CA
SH-SY5Y/TPEN	0.2	1.7	4.4	5.2	ChA > CAPE > RA > CA
SH-SY5Y/H_2_O_2_	1.1	6.2	3.9	>10	ChA > RA > CAPE > CA

## Data Availability

The data supporting the findings of this manuscript are available from the corresponding authors upon reasonable request.

## Supplementary Materials

The following supporting information can be downloaded at: https://www.mdpi.com/article/10.3390/biom16060920/s1, Figure S1. UV-vis absorption spectrum of 100 µM CAPE (200–800 nm). Figure S2. Quenching effect of 100 µM CAPE on ThT fluorescence in the absence of Aβ peptides (ThT-DMSO vs. ThT-CAPE).

## Abbreviations

The following abbreviations are used in this manuscript:

CA	Caffeic acid
CAPE	Caffeic acid phenethyl ester
ChA	Chicoric acid
RA	Rosmarinic acid
NLRP3	NLR family pyrin domain containing 3
H_2_O_2_	Hydrogen peroxide
TPEN	N,N,N′,N′-tetrakis(2-pyridinylmethyl)-1,2-ethanediamine
ROS	Reactive Oxygen Species
Aβ1–42	Amyloid-β1-42
pEAβ3–42	Pyroglutamate-modified Aβ3-42
ALZ-801	Valiltramiprosate
3-SPA	3-Sulfopropanoic acid
TEM	Transmission Electron Microscopy
