# Supplementary material for: Polyphenols Suppress Intracellular Zinc Deficiency-Induced ROS Production and NLRP3 Inflammasome Activation in Microglial and Neuronal Cells
Source: Biomolecules. 2026 Jun 21;16(6):920. doi: 10.3390/biom16060920 (PMC13296499; doi:10.3390/biom16060920)
Supplement: Supplementary file 1 [file biomolecules-16-00920-s001.zip › biomolecules-4342504-supplementary.pdf]

Figure S1. UV-vis absorption spectrum of 100 µM CAPE (200–800 nm).

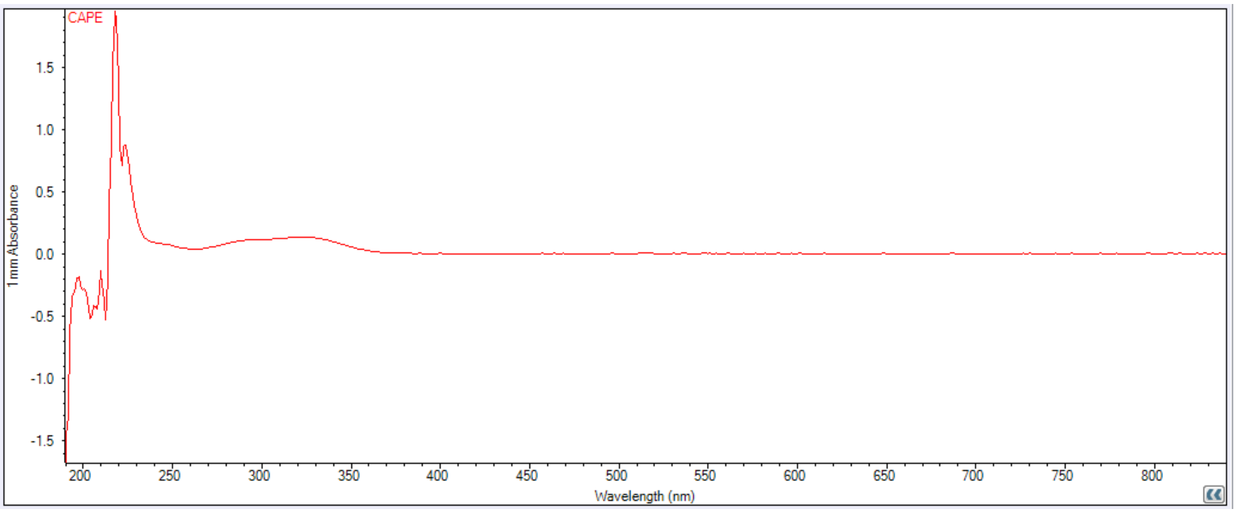

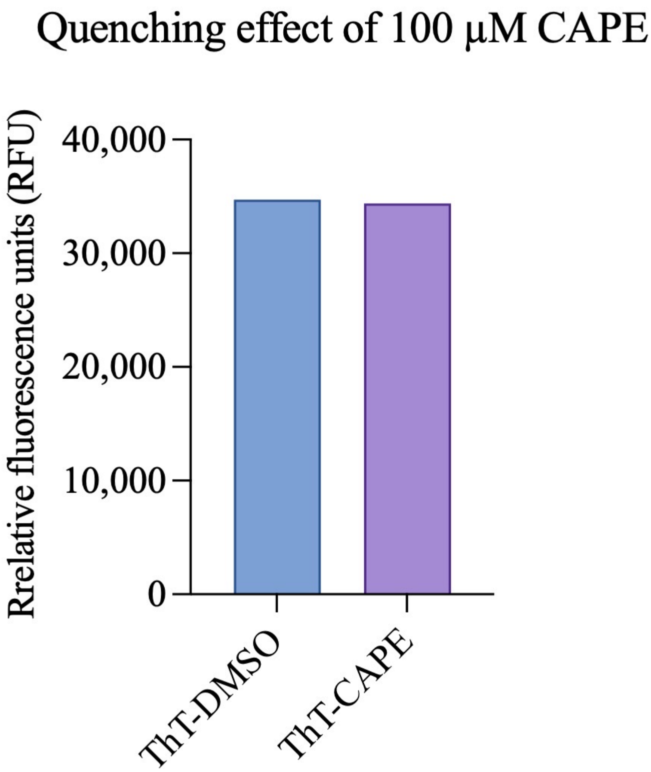

Figure S2. Quenching effect of 100 µM CAPE on ThT fluorescence in the absence of Aβ peptides (ThT-DMSO vs. ThT-CAPE).
